# Low serum total cholesterol levels predict inferior prognosis of patients with POEMS syndrome

**DOI:** 10.1007/s12672-024-00912-6

**Published:** 2024-03-04

**Authors:** Jue Zhang, Ting Zhang, Ye Yao, Xuxing Shen, Yuanyuan Jin, Run Zhang, Lijuan Chen

**Affiliations:** Department of Hematology, The First Affiliated Hospital of Nanjing Medical University, Jiangsu Province Hospital, Nanjing, 210029 China

**Keywords:** Low total cholesterol levels, Lipid profile, POEMS syndrome, Prognosis biomarker

## Abstract

Low serum cholesterol levels are associated with increased tumor morbidity and mortality. However, the relationship between serum lipid profile and POEMS syndrome (polyneuropathy, organomegaly, endocrinopathy, M-protein, skin changes) is still unclear. The aim of our study was to clarify the importance of the serum lipid profile in predicting the severity and prognosis of patients with POEMS syndrome. Forty-three patients with newly diagnosed POEMS syndrome admitted to the Department of Hematology of Jiangsu Provincial People's Hospital between August 2013 and February 2023 were selected. They had explicit serum lipid profiles. There were 27 males and 16 females with a median age of 54 years (range, 28–77 years). Survival curves were plotted using the Kaplan–Meier method, and comparisons between the two groups were performed using the log-rank test. The Cox proportional-hazards model examined risk factors associated with the prognosis of POEMS syndrome. Receiver-operator characteristic (ROC) curves assessed the predictive accuracy. 23 (53.5%) patients had low total cholesterol (TC) levels. Low levels of TC were concerned with unfavorable progression-free survival (PFS) (p = 0.007) and overall survival (OS) (p = 0.004), and at the same time, the low circulating TC concentration was an independent risk factor for PFS (*p* = 0.020) and OS (*p* = 0.011). Low TC values could improve the risk stratification, especially in high-risk patients. In conclusion, low serum TC levels may predict inferior prognosis in patients with POEMS syndrome; in future clinical application, low TC may be a reliable indicator of prognosis.

## Introduction

POEMS syndrome (polyneuropathy, organomegaly, endocrinopathy, M-protein, skin changes), also known as Crow-Fukase syndrome or Takatsuki syndrome, is a rare multi-system lesion with abnormal proliferation of plasma cells. The diagnostic criteria for POEMS syndrome are based on a combination of clinical and laboratory features: (1) polyneuropathy and monoclonal gammopathy; (2) one of the three other major criteria: (a) Castleman disease, (b) osteosclerosis, and (c) elevated serum VEGF level; (3) one of the six minor criteria: (a) organomegaly, (b) extravascular volume overload, (c) endocrinopathy, (d) skin changes, (e) papilledema, and (f) thrombocytosis and/or polycythemia [1]. The pathophysiology of POEMS syndrome is currently poorly understood. However, several studies have shown that patients with POEMS syndrome had significantly higher levels of vascular endothelial growth factor (VEGF), which has been associated with disease activity [2, 3]. The genetic or molecular risk factors that influence survival are not known. Therefore, we are striving to find a new and reliable indicator to assess the prognosis of patients with POEMS syndrome.

Patients with POEMS syndrome usually have severe dyslipidemia on arrival. Previous studies have reported that 37% of patients lost more than 5 kg of weight at the onset of the disease [1]. In a single-center clinical observation at Peking Union Medical University Hospital, 75% of patients had significant weight loss, most evident in a reduction in fat pads on the cheeks, and 36% of patients had hypocholesterolemia [4]. Since cholesterol is an essential component of cell membranes, the ability of tumor cells to increase cholesterol synthesis or accumulation is a prerequisite for their proliferation. Hypocholesterolemia has been found in patients with lung [5], gastrointestinal [6] and thyroid [7] cancers. Decreased cholesterol levels have been found in patients with hematological tumors [8], which is consistent with solid tumors such as chronic lymphocytic leukemia (CLL) [9, 10], acute lymphocytic leukemia [11], lymphoma [12, 13], and multiple myeloma [14], which may be due to increased use of cholesterol as an energy source by tumor cells. Cholesterol production, accumulation, demand and absorption were found to be increased in neoplastic cells, and this was particularly true for hematological tumors, which had a comparatively faster cell renewal rate than solid tumors. In addition, the concentration of HDL-C was lowered to minimize the loss of intracellular cholesterol pools during rapid tumor cell proliferation [8].

To date, however, there are only a few clinical studies on POEMS syndrome and hypocholesterolemia. The aim of the report was to clarify the relationship between the pre-diagnostic serum lipid profile and POEMS syndrome and the importance of hypocholesterolemia in predicting the severity and prognosis of patients with POEMS syndrome.

## Materials and methods

### Study population and ethics approval

43 patients with newly diagnosed POEMS syndrome who were admitted to the Department of Hematology of Jiangsu Provincial People's Hospital from August 2013 to February 2023 were selected. All met the diagnostic criteria for POEMS syndrome established by Dispenzieri et al. [1]. The Medical Research Ethics Committee of Jiangsu Provincial People's Hospital approved the study (No. 2020-SR-589) and informed consent was obtained from our patients.

### Risk stratification

According to the risk stratification method developed by Wang et al. [15], four basic criteria were followed: (a) age > 50 years, (b) pulmonary hypertension, (c) pleural effusion, and (d) glomerular filtration rate < 30 ml/min/1.73m^2^. The former three characteristics had a score of 1, the last a score of 2. Patients were divided into three groups based on the total score: Low, intermediate and high risk groups, which ranged from 0, 1, and 2 to 5, respectively.

### Data collection and definition of low total cholesterol level

Gender, age, splenomegaly, hepatomegaly, lymphadenopathy, edema, pleural effusion, ascites, pericardial effusion, diabetes mellitus, thyroid abnormalities, gonadal axis abnormalities, skin changes, sclerotic bone lesions, papilledema, pulmonary hypertension, and treatment regimens were obtained from the medical records as baseline clinical data. Laboratory data such as VEGF level, hemoglobin (Hb) level, platelet count, albumin level and creatinine were collected within 24 h of the first admission.

All patients were fasted for biochemical testing on the morning of the second day of hospitalization. A standard automated analyzer was used to determine serum lipid profiles, including total cholesterol (TC) (normal value range, 3.00–5.70 mmol/L), high-density lipoprotein cholesterol (HDL-C) (normal value range, 1.03–1.55 mmol/L), low-density lipoprotein cholesterol (LDL-C) (normal value range, 2.60–4.10 mmol/L), triglycerides (TG) (normal value range, 0.00–2.25 mmol/L) and lipoprotein (a) [Lp(a)] (normal value range, 0–1.017 µmol/L). Based on the manufacturer’s standard value, a low total cholesterol level was defined as TC < 3.00 mmol/L.

### Follow-up and survival evaluation

All patients were followed up by reviewing their inpatient or outpatient medical records, with a time limit until June 2023 and a median follow-up time of 43 (1–104) months. Follow-up events included progression-free survival (PFS) and overall survival (OS). PFS is defined as the time from diagnosis to disease progression, recurrence or death. OS is defined as the time from diagnosis to death or to the end of the follow-up period.

### Statistical analysis

All data were analyzed using SPSS 25.0 and GraphPad Prism 8.0, and data entry was double-checked. As all data were continuous variables with skewed distributions, they were represented by the median and interquartile range and compared by the rank sum test. Spearman correlation analysis was performed to examine the association between the therapies for POEMS syndrome and the lipid profile. Survival curves were plotted using the Kaplan–Meier method and comparisons between the two groups were performed using the log-rank test. The Cox proportional-hazards model examined risk factors associated with the prognosis of POEMS syndrome. Receiver operator characteristic (ROC) curves were calculated to assess prediction accuracy. Differences with a two-sided *p* < 0.05 were considered significant.

## Results

### Association between the lipid profile and baseline characteristics in patients with POEMS syndrome

43 patients with POEMS syndrome were selected who had clear serum lipid profiles at the time of initial diagnosis, with a median age of 54 years (range, 28–77 years). 23 (53.5%) patients had decreased TC levels, 37 (86.0%) patients had decreased HDL-C levels, 35 (81.4%) patients had decreased LDL-C levels, 5 (11.63%) patients had increased TG levels, and 13 (30.23%) patients had increased Lp(a) levels. In 32 (74.4%) of the patients, there was a simultaneous decrease in HDL-C and LDL-C.

The relationship between the lipid profile and baseline characteristics of 43 patients with POEMS syndrome is shown in Table 1. Synchronous decreases in TC, HDL-C and LDL-C were associated with lymphadenopathy and pleural effusion (*p* < 0.05) (Table 1). Furthermore, there was no apparent correlation between POEMS syndrome treatment regimens and the lipid profile [*r* = − 0.149, *p* = 0.341 for TC;* r* = − 0.276,* p* = 0.073 for TG; *r* = 0.142, *p* = 0.364 for HDL-C; *r* = − 0.070,* p* = 0.657 for LDL-C; *r* = 0.045, *p* = 0.774 for Lp(a)].

**Table 1:** Differences in serum cholesterol levels stratified by clinical characteristics in the POEMS syndrome patients

Clinical characteristics	Total [n, (%)]	TC [mmol/L, *M*(IQR)]	*p*	HDL-C [mmol/L, *M*(IQR)]	*p*	LDL-C [mmol/L, *M*(IQR)]	*p*	TG [mmol/L, *M*(IQR)]	*p*	Lp(a) [mg/L, *M*(IQR)]	*p*
Gender											
Male	27 (62.80)	2.56 (2.16–3.31)	0.183	0.65 (0.58–0.82)	0.715	1.57 (1.34–2.10)	0.039	1.08 (0.78–1.54)	0.085	185.00 (85.50–361.75)	0.443
Female	16 (37.20)	3.12 (2.35–4.51)		0.70 (0.60–0.88)		2.05 (1.74–2.82)		1.42 (1.13–2.52)		128.00 (79.00–354.00)	
Age											
< 65 years	33 (76.74)	3.03 (2.20–4.43)	0.249	0.67 (0.60–0.88)	0.402	1.87 (1.37–2.50)	0.470	1.17 (0.89–1.74)	0.745	151.00 (82.50–372.00)	0.419
≥ 65 years	10 (23.26)	2.51 (2.12–3.16)		0.66 (0.54–0.81)		1.70 (1.38–2.02)		1.08 (0.86–1.61)		128.00 (70.25–219.25)	
Polyneuropathy											
Yes	43 (100.00)	2.91 (2.21–3.67)	–	0.67 (0.58–0.83)	–	1.76 (1.37–2.37)	–	1.17 (0.89–1.63)	–	131.00 (81.00–354.00)	–
Organomegaly											
Splenomegaly											
Yes	29 (67.44)	3.03 (2.43–4.01)	0.204	0.65 (0.60–0.85)	0.959	1.87 (1.43–2.50)	0.577	1.36 (0.89–1.91)	0.209	185.00 (82.50–372.50)	0.228
No	14 (32.56)	2.42 (2.10–3.59)		0.72 (0.55–0.85)		1.76 (1.26–2.17)		1.09 (0.86–1.46)		123.50 (73.75–214.75)	
Hepatomegaly											
Yes	8 (18.60)	2.53 (2.16–3.58)	0.657	0.74 (0.59–0.93)	0.657	1.62 (1.29–2.50)	0.571	1.05 (0.68–1.59)	0.416	100.00 (61.00–348.25)	0.317
No	35 (81.40)	2.99 (2.31–4.35)		0.67 (0.58–0.83)		1.80 (1.48–2.37)		1.17 (0.90–1.76)		136.00 (84.00–372.00)	
Lymphadenopathy											
Yes	30 (69.77)	2.61 (2.18–3.29)	0.039	0.62 (0.57–0.74)	< 0.001	1.66 (1.33–2.10)	0.017	1.16 (0.69–1.43)	0.212	122.00 (77.75–200.50)	0.048
No	13 (30.23)	4.35 (2.36–4.70)		0.89 (0.70–1.05)		2.40 (1.68–2.94)		1.27 (0.95–1.78)		349.00 (101.50–725.00)	
Endocrinopathy											
Diabetes mellitus											
Yes	10 (23.26)	2.40 (1.89–3.42)	0.237	0.66 (0.60–0.86)	0.899	1.56 (1.29–2.02)	0.286	1.09 (0.73–2.01)	0.524	147.50 (69.50–412.75)	0.966
No	33 (76.74)	3.12 (2.35–4.01)		0.67 (0.58–0.85)		1.89 (1.44–2.39)		1.17 (0.91–1.60)		131.00 (81.00–363.00)	
Thyroid abnormalities											
Yes	33 (76.74)	2.65 (2.18–3.29)	0.041	0.65 (0.58–0.81)	0.149	1.75 (1.35–2.16)	0.133	1.16 (0.89–1.55)	0.788	128.00 (82.50–340.00)	0.854
No	10 (23.26)	4.13 (2.45–4.70)		0.80 (0.60–1.09)		2.20 (1.55–2.82)		1.31 (0.82–2.05)		158.00 (58.00–647.25)	
Gonadal axis abnormality											
Yes	31 (72.09)	2.66 (2.21–3.31)	0.446	0.65 (0.58–0.82)	0.355	1.76 (1.36–2.21)	0.243	1.17 (0.89–1.63)	1.000	131.00 (81.00–349.00)	0.495
No	12 (27.91)	3.02 (2.21–4.68)		0.70 (0.60–1.03)		1.93 (1.59–2.73)		1.17 (0.89–1.70)		156.50 (76.50–586.75)	
Monoclonal plasma cell dyscrasia											
+ IgA-L	21 (48.84)	3.03 (2.24–3.31)	0.072	0.64 (0.56–0.85)	0.462	1.87 (1.51–2.22)	0.038	1.17 (1.00–1.74)	0.884	125.00 (82.50–340.00)	0.499
IgA-L + IgG-L	5 (11.63)	2.51 (2.05–3.12)		0.65 (0.62–0.82)		1.50 (1.26–2.18)		1.17 (0.69–1.45)		122.00 (39.00–363.00)	
IgG-L	9 (20.93)	2.54 (1.67–4.00)		0.73 (0.59–0.79)		1.76 (1.10–2.54)		0.99 (0.82–2.41)		176.00 (67.00–749.50)	
L	4 (9.30)	2.48 (2.40–2.87)		0.67 (0.59–0.80)		1.55 (1.41–1.57)		1.13 (0.81–1.66)		95.00 (48.50–188.75)	
Others	4 (9.30)	4.61 (4.51–4.94)		0.91 (0.73–1.01)		2.88 (2.79–3.33)		1.30 (0.85–3.31)		278.50 (113.75–939.00)	
Skin changes											
Yes	35 (81.40)	2.66 (2.19–3.31)	0.132	0.64 (0.58–0.82)	0.026	1.75 (1.36–2.21)	0.149	1.17 (0.88–1.76)	0.613	122.00 (74.00–354.00)	0.274
No	8 (18.60)	3.91 (2.42–4.94)		0.82 (0.70–1.13)		2.20 (1.60–3.31)		0.98 (0.90–1.47)		156.00 (126.50–677.00)	
Extravascular volume overload											
Peripheral edema											
Yes	21 (48.84)	2.91 (1.89–3.23)	0.078	0.65 (0.61–0.80)	0.903	1.75 (1.22–2.04)	0.060	1.09 (0.75–1.45)	0.048	122.00 (77.00–298.00)	0.473
No	22 (51.16)	2.97 (2.45–4.57)		0.70 (0.57–0.88)		2.05 (1.54–2.65)		1.31 (0.95–2.01)		163.50 (80.50–372.00)	
Ascites											
Yes	17 (39.53)	2.32 (1.89–2.83)	0.001	0.65 (0.62–0.79)	0.700	1.48 (1.22–1.75)	0.001	0.99 (0.75–1.17)	0.006	136.00 (92.00–286.50)	0.980
No	26 (60.47)	3.29 (2.50–4.57)		0.68 (0.58–0.96)		2.09 (1.70–2.78)		1.45 (1.05–2.05)		129.50 (73.75–396.25)	
Pleural effusion											
Yes	23 (53.49)	2.49 (1.90–3.22)	0.007	0.61 (0.58–0.73)	0.006	1.54 (1.27–2.08)	0.003	1.15 (0.89–1.76)	0.874	119.00 (73.00–198.00)	0.128
No	20 (46.51)	3.26 (2.55–4.68)		0.79 (0.67–1.03)		2.05 (1.74–2.83)		1.19 (0.79–1.55)		196.50 (85.75–550.00)	
Pericardial effusion											
Yes	27 (62.79)	2.65 (2.31–3.23)	0.145	0.64 (0.58–0.76)	0.042	1.75 (1.36–2.08)	0.116	1.17 (0.96–1.76)	0.482	131.00 (81.00–354.00)	0.990
No	16 (37.21)	3.91 (2.18–4.68)		0.85 (0.64–1.02)		2.30 (1.44–2.80)		1.17 (0.73–1.58)		156.50 (70.50–366.25)	
Bone lesions											
Yes	15 (34.88)	2.56 (1.90–3.23)	0.262	0.65 (0.60–0.77)	0.819	1.54 (1.11–1.89)	0.037	1.14 (0.92–1.76)	0.858	100.00 (81.00–208.00)	0.346
No	28 (65.12)	3.08 (2.33–4.47)		0.68 (0.58–0.88)		2.04 (1.54–2.54)		1.19 (0.88–1.61)		163.50 (81.75–367.50)	
Papilledema											
Yes	7 (16.28)	2.32 (1.89–3.03)	0.137	0.63 (0.58–0.65)	0.166	1.48 (1.34–1.99)	0.104	1.09 (0.88–1.17)	0.356	122.00 (73.00–372.00)	0.936
No	36 (83.72)	3.06 (2.33–4.47)		0.71 (0.59–0.89)		1.84 (1.54–2.54)		1.28 (0.89–1.73)		133.50 (81.00–352.75)	
Pulmonary hypertension											
Yes	11 (25.58)	2.56 (1.89–3.31)	0.450	0.61 (0.58–0.68)	0.230	1.55 (0.92–2.21)	0.219	1.17 (0.89–1.97)	0.732	119.00 (73.00–131.00)	0.098
No	32 (74.42)	3.01 (2.24–4.18)		0.72 (0.60–0.89)		1.88 (1.40–2.54)		1.17 (0.89–1.54)		180.50 (81.75–372.75)	
VEGF elevation											
Yes	39 (90.70)	2.66 (2.21–3.67)	0.856	0.65 (0.58–0.82)	0.083	1.76 (1.37–2.37)	0.825	1.17 (0.89–1.63)	0.762	125.00 (79.00–349.00)	0.083
No	4 (9.30)	3.11 (1.91–4.33)		0.82 (0.74–1.01)		1.73 (1.07–2.58)		1.18 (0.70–1.93)		408.50 (154.00–723.75)	
Hb											
< 130 g/L	30 (69.77)	2.55 (2.19–3.34)	0.167	0.68 (0.61–0.85)	0.380	1.76 (1.36–2.18)	0.300	1.10 (0.75–1.45)	0.002	156.00 (83.25–372.00)	0.395
≥ 130 g/L	13 (30.23)	3.30 (2.56–4.43)		0.61 (0.57–0.82)		2.10 (1.54–2.57)		1.76 (1.16–2.46)		119.00 (70.00–267.00)	
PLT											
< 350 *10^9^/L	31 (72.09)	2.51 (2.17–3.30)	0.007	0.65 (0.58–0.80)	0.157	1.57 (1.34–2.08)	0.004	1.09 (0.75–1.52)	0.026	136.00 (74.00–349.00)	0.659
≥ 350 *109/L	12 (27.91)	3.82 (3.05–4.65)		0.80 (0.61–0.92)		2.35 (1.82–2.89)		1.50 (1.15–2.61)		112.50 (81.75–664.50)	
Albumin											
< 35 g/L	37 (86.05)	2.66 (2.20–3.48)	0.277	0.65 (0.58–0.81)	0.031	1.75 (1.37–2.22)	0.327	1.16 (0.89–1.60)	0.720	131.00 (81.00–340.00)	0.771
≥ 35 g/L	6 (13.95)	3.89 (2.35–4.63)		0.92 (0.66–1.13)		2.23 (1.61–2.50)		1.43 (0.84–1.91)		250.50 (49.00–540.00)	
Creatinine											
< 177 umol/L	38 (88.37)	3.01 (2.29–3.84)	0.290	0.66 (0.58–0.88)	0.927	1.84 (1.47–2.38)	0.449	1.19 (0.92–1.66)	0.273	129.50 (80.50–358.50)	0.811
≥ 177 umol/L	5 (11.63)	2.32 (1.98–3.60)		0.68 (0.57–0.82)		1.48 (1.35–2.29)		0.88 (0.69–2.51)		185.00 (77.50–494.50)	
Risk stratification											
Low-risk	4 (9.30)	4.66 (4.42–4.70)	0.014	0.95 (0.79–1.13)	0.078	2.71 (2.45–2.93)	0.014	1.77 (1.04–2.62)	0.471	567.50 (154.00–930.00)	0.129
Medium-risk	20 (46.51)	3.01 (2.31–3.30)		0.66 (0.58–0.87)		1.88 (1.40–2.19)		1.17 (0.76–1.51)		191.50 (75.25–367.50)	
High-risk	19 (44.19)	2.54 (1.89–3.31)		0.65 (0.58–0.80)		1.55 (1.11–1.99)		1.15 (0.89–1.76)		125.00 (81.00–176.00)	
Hypertensive											
Yes	16	2.55 (2.20–3.12)	0.196	1.18 (1.01–1.73)	0.400	0.68 (0.59–0.79)	0.734	1.56 (1.29–1.85)	0.039	92.00 (68.50–166.00)	0.035
No	27	3.22 (2.21–4.51)		1.16 (0.75–1.54)		0.67 (0.58–0.93)		2.08 (1.54–2.60)		185.00 (90.00–373.00)	
Cardiac disease											
Yes	4	2.32 (1.88–5.77)	0.479	1.04 (0.67–1.62)	0.479	0.73 (0.62–1.14)	0.454	1.64 (1.39–3.55)	0.920	494.50 (127.00–795.25)	0.244
No	39	2.99 (2.31–3.67)		1.17 (0.90–1.63)		0.67 (0.58–0.83)		1.80 (1.37–2.37)		128.00 (81.00–349.00)	
Renal disease											
Yes	7	2.49 (2.17–4.51)	0.640	1.43 (0.75–2.77)	0.885	0.68 (0.60–0.82)	0.987	1.73 (1.29–2.77)	0.617	90.00 (84.00–198.00)	0.448
No	36	3.01 (2.31–3.66)		1.17 (0.91–1.61)		0.66 (0.58–0.86)		1.84 (1.49–2.33)		133.50 (79.50–372.00)	
Lipid-lowering drugs											
Yes	4	2.41 (1.74–2.53)	0.109	1.22 (0.97–1.50)	0.984	0.72 (0.51–0.79)	0.856	1.76 (1.12–1.93)	0.505	105.00 (32.75–166.00)	0.227
No	39	3.03 (2.21–4.35)		1.17 (0.88–1.76)		0.65 (0.58–0.87)		1.80 (1.37–2.40)		131.00 (81.00–372.00)	
Treatments											
RD	26 (60.47)	3.01 (2.37–4.39)	0.482	0.65 (0.58–0.76)	0.833	1.81 (1.50–2.59)	0.270	1.19 (1.06–1.83)	0.268	129.50 (83.25–268.75)	0.277
VD	5 (11.63)	2.49 (2.04–2.83)		0.80 (0.60–0.83)		1.36 (1.11–1.78)		0.96 (0.77–1.33)		125.00 (59.00–187.00)	
CD	4 (9.30)	3.51 (1.91–4.94)		0.76 (0.50–0.99)		2.40 (1.50–3.31)		1.20 (0.78–1.97)		710.00 (220.00–1036.50)	
Others	8 (18.60)	2.87 (1.95–3.29)		0.75 (0.60–1.02)		1.83 (1.29–2.18)		1.03 (0.60–1.74)		225.00 (35.00–442.75)	

TC, total cholesterol; HDL-C, high-density lipoprotein cholesterol; LDL-C, low-density lipoprotein cholesterol; TG, triglycerides; Lp(a), lipoprotein (a); Hb, hemoglobin; PLT, platelet; RD, lenalidomide and dexamethasone; VD, bortezomib and dexamethasone; CD, cyclophosphamide and dexamethasone

### Prognostic value of the serum lipid profile in POEMS syndrome

**Fig. 1:**
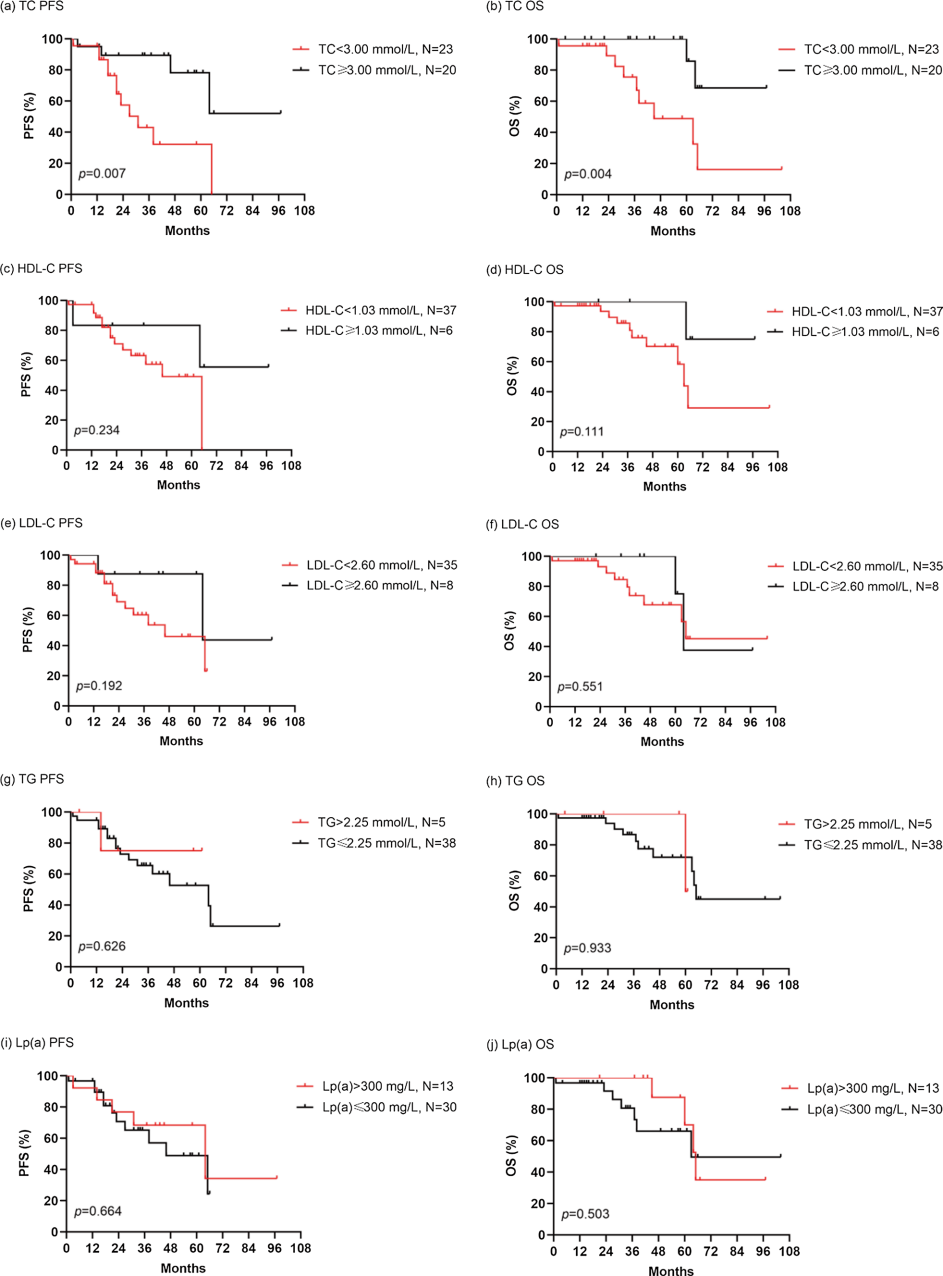
Kaplan–Meier survival curves of the POEMS syndrome patients stratified by serum lipid profile (**a**, **b**) Kaplan–Meier curves for PFS (**a**) and OS (**b**) of patients with different TC levels. **c**, **d** Kaplan–Meier curves for PFS (**c**) and OS (**d**) of patients with different HDL-C levels. **e**, **f** Kaplan–Meier curves for PFS (**e**) and OS (**f**) of patients with different LDL-C levels. **g**, **h** Kaplan–Meier curves for PFS (**g**) and OS (**h**) of patients with different TG levels. **i**, **j** Kaplan–Meier curves for PFS (**i**) and OS (**j**) of patients with different Lp (**a**) levels. TC, total cholesterol; HDL-C, high-density lipoprotein cholesterol; LDL-C, low-density lipoprotein cholesterol; TG, triglycerides; Lp (**a**), lipoprotein (**a**); PFS, progression free survival; OS, overall survival

The median follow-up time was 43 months (range, 1–104) to June 30, 2023, with 16 (37.2%) patients experiencing disease progression and 11 (25.6%) patients dying. Patients with low TC had inferior PFS (31 months vs. NR, p = 0.007) (Fig. 1a). Similarly, low TC was clearly associated with shorter OS (45 months vs. NR, p = 0.004) (Fig. 1b). Nevertheless, no obvious difference was observed in survival outcomes stratified by HDL-C, LDL-C, TG and Lp(a) (p > 0.05) (Fig. 1c–j). Survival analysis showed that patients with POEMS syndrome who had reduced TC levels had a worse prognosis.

The Cox regression analysis examined the risk factors associated with the prognosis of POEMS syndrome. The variables with a *p* < 0.15 in the univariable regression analysis were further included in the multivariable regression analysis. Age ≥ 65 years (*HR* = 3.107, 95% *CI* 1.108–8.715, *p* = 0.031) and TC < 3.00 mmol/L (*HR* = 4.124, 95% *CI* 1.251–13.595, *p* = 0.020) were independent risk factors for PFS. Similarly, age ≥ 65 years (*HR* = 4.433, 95% *CI* 1.265–15.531, *p* = 0.020) and TC < 3.00 mmol/L (*HR* = 7.887, 95% *CI* 1.594–39.034, *p* = 0.011) were also independent risk factors for OS (Table 2).

**Table 2:** Univariable and multivariable analyses of PFS and OS in the POEMS syndrome patients

Variables	PFS				OS			
	Univariable Analyses		Multivariable Analyses		Univariable Analyses		Multivariable Analyses	
	*HR* (95% *CI*)	*p*	*HR* (95% *CI*)	*p*	*HR* (95% *CI*)	*p*	*HR* (95% *CI*)	*p*
Male	2.275 (0.728–7.109)	0.158			1.948 (0.512–7.420)	0.328		
Age ≥ 65 years	3.514 (1.267–9.743)	0.016	3.107 (1.108–8.715)	0.031	4.187 (1.258–13.937)	0.02	4.433 (1.265–15.531)	0.020
Organomegaly	1.787 (0.360–8.869)	0.477			2.371 (0.298–18.873)	0.415		
Edocrinopathy	2.222 (0.286–17.277)	0.445			1.573 (0.198–12.493)	0.668		
Skin changes	0.670 (0.226–1.982)	0.469			0.615 (0.174–2.172)	0.45		
Extravascular volume overload	3.523 (0.772–16.070)	0.104	1.930 (0.413–9.027)	0.403	2.221 (0.474–10.411)	0.311		
Bone lesions	0.726 (0.200–2.641)	0.627			0.441 (0.055–3.522)	0.44		
VEGF elevation	0.358 (0.078–1.633)	0.185			1.163 (0.148–9.165)	0.886		
TC < 3.00 mmol/L	4.393 (1.376–14.027)	0.012	4.124 (1.25–13.595)	0.020	7.200 (1.54–33.666)	0.012	7.887 (1.594–39.034)	0.011
HDL-C < 1.03 mmol/L	2.628 (0.515–13.41)	0.245			4.826 (0.586–39.732)	0.143	0.917 (0.056–15.024)	0.952
LDL-C < 2.60 mmol/L	2.601 (0.582–11.624)	0.211			1.594 (0.340–7.460)	0.554		
TG > 2.25 mmol/L	0.607 (0.079–4.668)	0.632			0.914 (0.111–7.506)	0.933		
Lp(a) > 300 mg/L	0.792 (0.273–2.294)	0.667			0.657 (0.190–2.269)	0.506		

TC, total cholesterol; HDL-C, high-density lipoprotein cholesterol; LDL-C, low-density lipoprotein cholesterol; TG, triglycerides; Lp(a), lipoprotein (a); VEGF, vascular endothelial growth factor; PFS, progression-free survival; OS, overall survival

### Subgroup analysis of low serum total cholesterol levels in POEMS syndrome

**Fig. 2:**
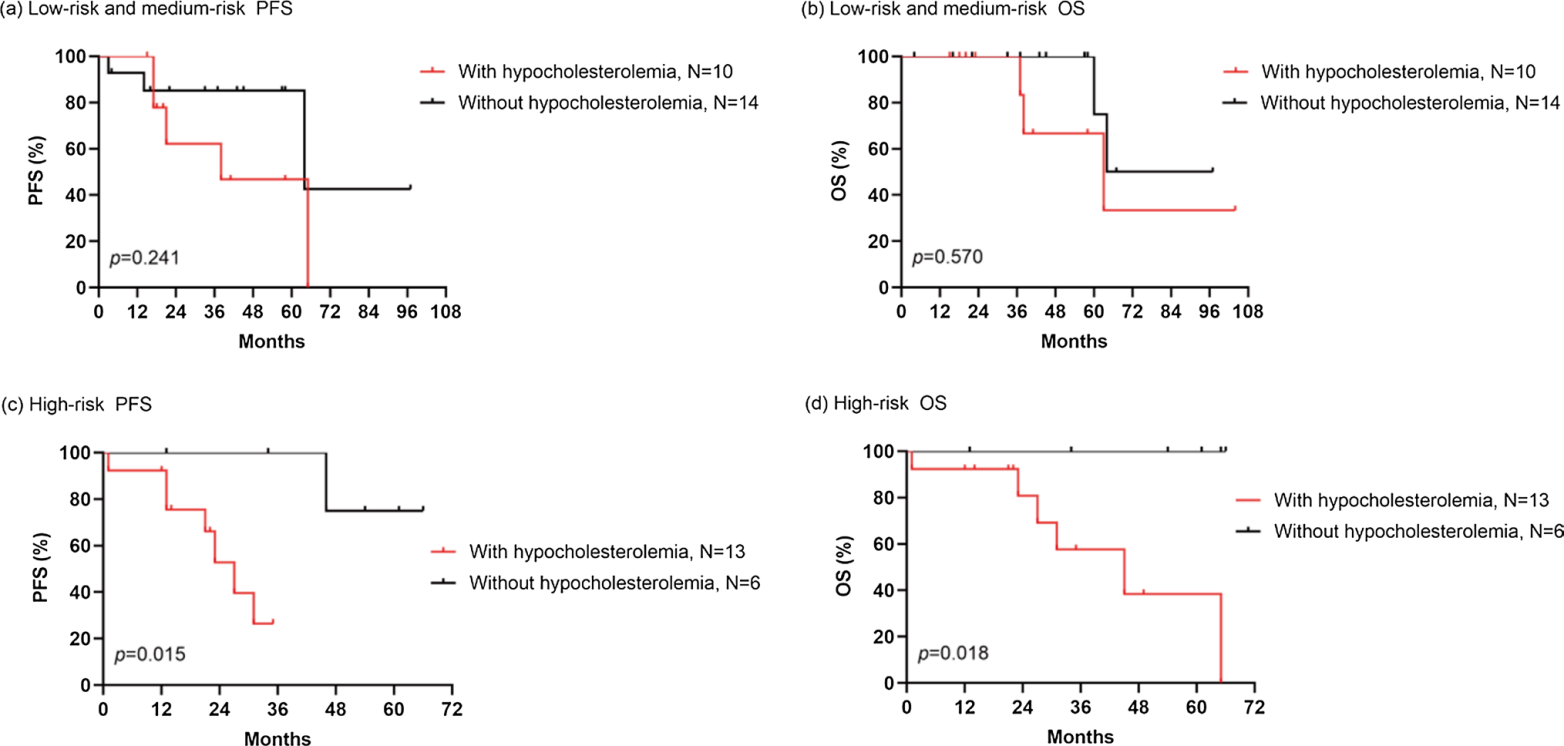
Subgroup survival analyses of risk stratification for PFS and OS (**a**, **b**) Kaplan–Meier curves for PFS (**a**) and OS (**b**) of patients in high-risk group with low total cholesterol levels or non-low total cholesterol levels. **c**, **d** Kaplan–Meier curves for PFS (**c**) and OS (**d**) of patients in low-risk or medium-risk groups with low total cholesterol levels or non-low total cholesterol levels. PFS, progression free survival; OS, overall survival

For the subgroup analysis for risk stratification, we divided our 43 POEMS syndrome patients into two categories: 24 into the low- or intermediate-risk groups and 19 into the high-risk group. Survival analysis was performed based on specific prognostic risk. Individuals with reduced TC concentration had significantly inferior PFS or OS than those with normal TC concentration in the high-risk group (27 months *vs.* NR, *p* = 0.015 for PFS; 45 months *vs.* NR, *p* = 0.018 for OS) (Fig. 2c–d). However, there was no apparent difference in PFS or OS between those with low and non-low TC in the low and intermediate risk groups (*p* > 0.05) (Fig. 2a, b).

### ROC analyses of TC for the prediction of PFS and OS

**Fig. 3:**
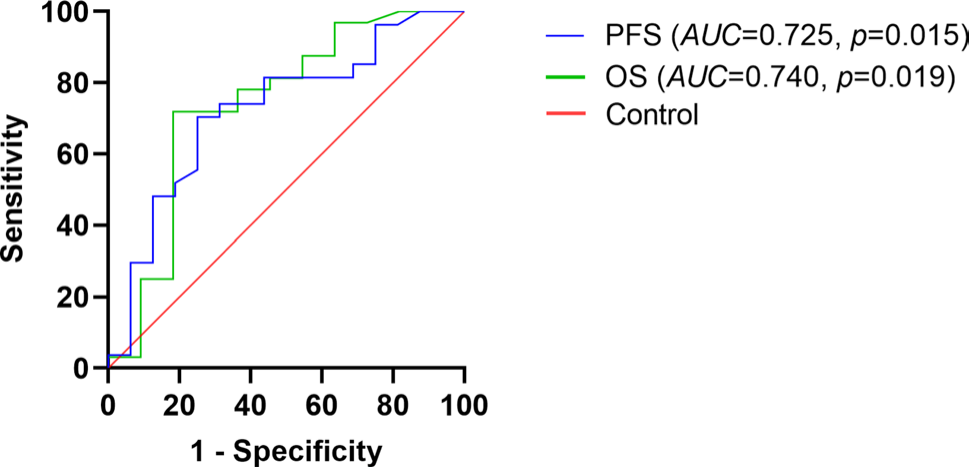
Receiver operating characteristics (ROC) curve analysis using TC for predicting PFS and OS of POEMS syndrome TC yielded an AUC (the areas under the ROC curve) of 0.725 (95% *CI* 0.564–0.886, *p*= 0.015) for PFS, and an AUC of 0.740 (95% *CI* 0.546–0.934, *p* = 0.019) for OS. TC, total cholesterol; PFS, progression free survival; OS, overall survival

To evaluate the predictive power of TC for PFS and OS, we plotted the ROC curve (Fig. 3). The results showed that the AUC of TC for PFS was 0.725 (95% CI: 0.564–0.886, p = 0.015) and for OS was 0.740 (95% CI: 0.546–0.934, p = 0.019). For the prediction of PFS and OS, 2.66 mmol/L and 2.55 mmol/L, respectively, were the ideal TC cut-off values.

## Discussion

The prognostic significance of hypocholesterolemia in POEMS syndrome is being examined for the first time in this study. Cholesterol, as a highly reproducible, easy to detect, and affordable laboratory indicator, may be a reliable candidate indicator for predicting the prognosis of POEMS syndrome, even though the molecular mechanisms that link cholesterol to cancer remain elusive.

Cholesterol is known to play an important role in the maintenance of cell membranes and the regulation of membrane fluidity and function, including transmembrane signaling and cell adhesion to the extracellular matrix. It is also known that cancer cell proliferation is dependent on either the de novo synthesis of cholesterol in the endoplasmic reticulum or the uptake of cholesterol from the bloodstream through receptor-mediated endocytosis of low-density lipoproteins (LDL) [16]. Tumor cells preferentially meet their high cholesterol requirements by increasing endogenous cholesterol synthesis through upregulation of HMG-CoA reductase (3-hydroxy-3-methylglutaryl coenzyme A reductase) and increased expression of the LDL receptor [17, 18]. Therefore, a low serum TC level may be a sign of tumor progression. Previous epidemiologic studies have also shown that low serum cholesterol levels are associated with higher cancer incidence and higher cancer-related mortality. This could be explained by the relationship between the immune system and cholesterol metabolism. There are several reports that a low serum TC level is associated with an impaired immune system. Men with hypocholesterolemia have been found to have significantly fewer circulating lymphocytes, T cells and CD8 cells than men with hypercholesterolemia [19]. Bensinger et al. reported that cellular accumulation of cholesterol is essential for the activation and proliferation of CD4þ T cells [20]. Cholesterol binds directly to the a-chain of the T cell receptor and thus regulates nanoclustering and activation of the receptor. In addition, T cell activation triggers a simultaneous suppression of the liver X receptor pathway for cholesterol transport and an induction of the sterol regulatory element- binding protein pathway for cholesterol synthesis. However, even after T cell activation, T cell proliferation is prevented if cholesterol accumulation is not achieved. Therefore, persistently low serum cholesterol levels may impair cell-mediated immunity and lead to immune escape and cancer progression. Thus, low serum cholesterol might be associated with tumor development or progression.

Hypocholesterolemia was found in hematological tumors. Kuliszkiewicz-Janus et al. discovered a simultaneous reduction in TC, HDL-C and LDL-C concentrations during disease activity of acute leukemia and non-Hodgkin's lymphoma (NHL) [21]. Gao et al. identified the lipid profiles in CLL and diffuse large B-cell lymphoma (DLBCL), which manifested as reduced concentrations of TC, HDL-C and LDL-C [10, 13]. Yavasoglu et al. also discovered that newly diagnosed CLL and multiple myeloma (MM) patients had significantly lower TC, HDL-C and LDL-C levels [9, 14]. Our results supported the previously mentioned finding that the majority of patients with POEMS syndrome had lower TC, HDL-C and LDL-C levels. An inverse relationship between HDL-C and NHL was also observed by Lim et al. according to which the risk of NHL decreased by 15% with every 5 mg/dL increase in HDL-C level [12]. Shor et al. also discovered that the risk of hematological malignancy decreased by 2.4% for every 1 mg/dL increase in LDL-C levels [22].

Our study discovered that lower TC levels at diagnosis were correlated with poorer PFS and OS in patients with POEMS syndrome, and at the same time, low TC in the serum was an independent risk factor for both PFS and OS. In line with our findings on tumor prognosis, Parsa N et al. showed in a prospective cohort study that a low serum cholesterol level was significantly correlated with a higher risk of overall cancer mortality [23]. In DLBCL patients, Gao et al. showed that TC was linked to poor PFS and OS [13]. Additionally, CLL patients with hypocholesterolemia also had a worse time to first treatment (TTFT) and cancer-specific survival (CSS) [10]. Serum cholesterol has therefore been proposed as a prognostic indicator for POEMS syndrome. We believe that it is a good idea for patients with POEMS syndrome to undergo serum lipid testing on initial admission, which can be easily performed in all hospitals, including community hospitals. Special attention should be paid to patients with low TC because of their potentially worse disease outcomes.

Patients with concomitant low TC levels had poorer OS in the high-risk group compared with those with normal TC levels, but there was no discernible difference in the low- or intermediate-risk groups. We speculated that decreased TC levels may serve as a predictive factor for mortality in patients in the high-risk group, as it may further identify the high-risk patients in the high-risk group. However, it would be inappropriate to directly use our results as a new criterion for risk stratification of patients because we need larger data to re-examine our results and validate them in different centers.

The cut-off value for TC constructed from the ROC curves was slightly lower than 3 mmol/L in this study for the following possible reasons. First, the level of TC may vary according to ethical or geographical differences [24]. Second, 3 mmol/L is the lower limit of the normal range for a healthy population, and our cut-off value was derived from patients with POEMS syndrome who had relatively low TC levels. Thus, further large cohort studies and external validation studies should be conducted to determine the exact serum TC concentration that has the highest prognostic value in patients with POEMS syndrome.

The present study was subject to several limitations. First, it was a retrospective study at a single center with a single participant and a small sample size, which may lead to selection and sampling bias. Even if we control the balance for measured factors with a strict statistical correction, there may be unmeasured confounders, such as nutritional status, chronic liver disease, and critical illness status, which are also associated with low TC. Second, the wide range of diagnosis timing might lead to large heterogeneity. Third, our analysis is based on retrospective data from the Asian population, which may also limit the generalizability of our findings. Larger clinical studies, especially prospective studies, are required to further define the prognostic impact of lowered TC levels in individuals with POEMS syndrome.

In summary, patients with POEMS syndrome who had a low serum TC concentration were more likely to have an unfavorable outcome. A low TC level could allow further identification of high-risk cases in the high-risk group and thus improve risk stratification for POEMS syndrome. Given that lipid levels could be easily monitored and are reproducible, a low TC level might be an ideal prognostic marker for future clinical practice in POEMS syndrome.

## Data Availability

The data that support the findings of this study are available from the corresponding author upon reasonable request.
